# Clergyman's Sore Throat

**Published:** 1889-12-28

**Authors:** 


### SURGERY.

### CLERGYMAN'S SORE THROAT.

The *Bristol Medico-Chirurgical Journal* for November contains an interesting article on "Chronic Pharyngitis," or clergyman's sore throat, from the pen of Dr. Watson Williams, physician in charge of the throat department of the Bristol Royal Infirmary. Clergyman's sore throat is one of the most intractable conditions met with, and is almost invariably associated with a relaxed uvula. Cases of the kind may be conveniently classed under two heads: those in which the uvula and soft palate are merely relaxed, and those in which there is chronic congestion and hypertrophy. In the simpler cases, where there is merely relaxation of the uvula and soft palate, the symptoms are mainly impairment of the quality and strength of the voice. But elongation of the uvula may lead to paresis, which prevents the proper and necessary movements when the voice is used. If the voice is still used the constant strain may lead to congestion and hypertrophy. In a marked case the patient complains of hawking, with a feeling of some foreign body in the throat, which cannot be coughed up, and sleep is prevented. The emaciation, cough, and expectoration often streaked with blood, have been mistaken for pulmonary phthisis. A variety of causes lead to this elongation of the uvula, a common one being using the voice during catarrhal attacks. The resulting paresis is probably due to peripheral neuritis of the posterior palatine nerve at its exit from the posterior palatine canal. In treating cases of relaxed uvula it is well to give local astringents a fair trial. When local applications fail the uvula should be partially removed. Dr. Williams has always obtained good results by ablation of the uvula. Now we believe there are other causes not mentioned by the author which produce clergyman's sore throat. One is straining the voice before it has been well trained, which many young clergymen do. Another is wearing a stiff collar, which presses on the throat when the head is bent, and produces constriction of the parts. As a necessity of their position, clergymen, moreover, look downwards. Barristers, who look upwards, and do not wear tight stiff stocks, rarely suffer from the complaint; although, like priests, they are in crowded heated places. But in many instances clergyman's sore throat is simply evidence of constitutional debility or poor living and hard work. As Dr. Williams observes, the use of tobacco must not be forgotten as a possible factor in some cases. We are rather inclined to think that the author relies too much on division of the uvula as a curative measure. If the case is one of simple elongation it may succeed, but if the parts are much congested and hypertrophied the only effectual remedies are rest for weeks, sometimes for months, improvement of the general health, then gradually bringing the voice into play, while avoiding the band-like stock, and also avoiding looking down when in the pulpit. The beard should be allowed to grow, and sudden transitions from cold to heated atmosphere, and the reverse, should be avoided as much as possible.

